# Considerations for a Respiratory Syncytial Virus Vaccine Targeting an Elderly Population

**DOI:** 10.3390/vaccines9060624

**Published:** 2021-06-09

**Authors:** Laura M. Stephens, Steven M. Varga

**Affiliations:** 1 Interdisciplinary Graduate Program in Immunology, University of Iowa, Iowa City, IA 52242, USA; laura-stephens@uiowa.edu; 2 Department of Microbiology and Immunology, University of Iowa, Iowa City, IA 52242, USA; 3 Department of Pathology, University of Iowa, Iowa City, IA 52242, USA

**Keywords:** respiratory syncytial virus, RSV, vaccine, elderly, immune senescence, aged, defect

## Abstract

Respiratory syncytial virus (RSV) is most commonly associated with acute lower respiratory tract infections in infants and children. However, RSV also causes a high disease burden in the elderly that is often under recognized. Adults >65 years of age account for an estimated 80,000 RSV-associated hospitalizations and 14,000 deaths in the United States annually. RSV infection in aged individuals can result in more severe disease symptoms including pneumonia and bronchiolitis. Given the large disease burden caused by RSV in the aged, this population remains an important target for vaccine development. Aging results in lowered immune responsiveness characterized by impairments in both innate and adaptive immunity. This immune senescence poses a challenge when developing a vaccine targeting elderly individuals. An RSV vaccine tailored towards an elderly population will need to maximize the immune response elicited in order to overcome age-related defects in the immune system. In this article, we review the hurdles that must be overcome to successfully develop an RSV vaccine for use in the elderly, and discuss the vaccine candidates currently being tested in this highly susceptible population.

## 1. Introduction

Respiratory syncytial virus (RSV) is a negative-sense, single-stranded RNA virus of the *Pneumoviridae* family [1]. RSV replicates efficiently in the lungs, resulting in an acute respiratory tract infection characterized by mild rhinorrhea symptoms, or coughing and wheezing [2,3]. Despite virtually all children being infected with RSV by three years of age, individuals remain susceptible to repeated infections throughout adulthood. Thus, natural exposure to RSV affords incomplete, long-term immunity [4,5]. Additionally, high-risk groups are susceptible to more serious symptoms including pneumonia and increased mortality upon reinfection [6,7]. The only FDA-approved treatment for RSV infection is palivizumab, a monoclonal antibody directed against the RSV fusion (F) protein. Palivizumab is only recommended for prophylactic use in sero-negative high-risk pediatric patients as it is ineffective when administered as a treatment post RSV-infection [8]. Additionally, palivizumab is expensive and reduces the rate of severe RSV-associated hospitalization by only ~50% when multiple doses are administered prior to an RSV infection [9,10]. While it is an active area of research, there is currently no licensed vaccine for RSV.

RSV has been primarily recognized as the leading cause of respiratory infections in children and infants [5,11]. However, RSV is also an important viral pathogen among aging adults. In the United States, the estimated mortality rate of RSV in elderly adults exceeds that of children. While RSV causes approximately 500 deaths each year in children less than 5 years old, it causes an estimated 14,000 deaths annually in adults >65 years of age [12,13,14,15]. Additionally, the total hospitalization cost of patients with RSV-related symptoms is estimated to be $150–680 million annually, with the highest burden in those aged >65 years [16,17]. Elderly individuals are also at an increased risk of developing more severe symptoms including pneumonia and bronchiolitis following RSV infection, resulting in prolonged hospitalizations [3,18,19]. Given that the elderly represents a substantial portion of RSV burden, the development of a vaccine targeting this important population is highly desirable. However, the diminished immune responsiveness exhibited by older adults makes it more difficult to induce a robust immune response following vaccination. Here, we review the current literature on RSV vaccines for the elderly, and discuss important factors that must be considered for generating the most efficacious vaccine formulation. 

## 2. The Aged Immune System and RSV

There are a number of contributing factors responsible for the increased burden of RSV in the elderly population. The presence of underlying co-morbidities including chronic cardiac or pulmonary disease, diabetes, and severe immunosuppression predispose elderly individuals to RSV infection. One study tracking adult patients over four consecutive winter seasons found that RSV infection occurred in 3–7% of healthy elderly adults and 4–10% of high-risk patients [13]. Additionally, pre-existing conditions predispose elderly adults to more severe outcomes following RSV infection [3,20,21,22]. The hospitalization admission rate for RSV-associated community-acquired pneumonia increased with age, with an average incidence of 0.1/10,000 people per year for those 18–49 years of age, 0.8/10,000 (50–64 years of age), 2.5/10,000 (65–79 years of age), and 5/10,000 (>80 years of age) [23]. The incidence of RSV-associated severe disease in this population is enhanced compared to other respiratory infections, as adults >60 years old hospitalized with RSV exhibited an increased rate of developing pneumonia, chronic bronchitis, and increased mortality compared to those hospitalized with an influenza infection [24]. Aging also reduces pulmonary function and causes a weakening of epithelial integrity that results in a compromised ability of aged individuals to fight off respiratory pathogens [25,26]. Thus, RSV disproportionally impacts elderly individuals, resulting in an increased likelihood of severe disease following infection.

### 2.1. Immune Senescence

**Figure 1:**
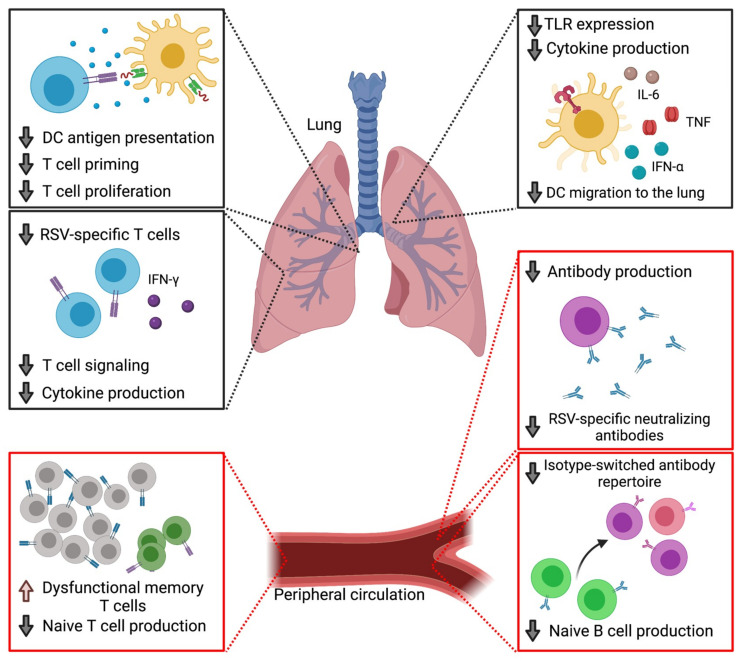
Key features of immune senescence. With age, the production of new T cells entering the periphery declines while pre-existing memory T cells progress into dysfunction. Aged T cells are also impaired in their signaling capacity and are less responsive to in vitro stimulation. Following RSV infection, aged individuals exhibit a reduced frequency of RSV-specific T cells and impaired functional capacity as demonstrated by reduced IFN-γ production. Aged dendritic cells produce lower quantities of many proinflammatory cytokines including IL-6, TNF, and IFN-α in response to various TLR stimulation. They also demonstrate a reduced capacity to prime naïve CD4 and CD8 T cells as measured by proliferation and cytotoxicity. Aging also results in delayed migration of DCs into the lung and lung-draining lymph nodes following viral infection. The aged humoral immune response is characterized by a decrease in the production of naïve B cells, as well as a reduction in class-switch recombination and somatic hypermutation, resulting in a reduced diversity of the B cell repertoire. RSV-specific antibody responses including serum neutralizing antibody titers decline with age. Created with BioRender.com. Accessed on 3 June 2021.

Older adult populations exhibit a progressive decline in immune function, known as immune senescence (Figure 1). Characterized by diminished innate and adaptive immune responses, this weakened immune responsiveness increases the susceptibility to numerous bacterial and viral infections including RSV [27,28]. Murine studies have demonstrated that aging alters the steady-state gene profile of lung tissue, with the upregulation of genes associated with immune-modulation and activation including *Cxcl9*, *Cxcr6*, *Gata3*, and *Cybb* [29]. This elevated basal inflammatory state, termed inflammaging, may negatively impact an individual’s response to both infection and vaccination [30,31,32]. This is supported by studies demonstrating enhanced pathology and an inability to rapidly clear virus in aged animals following RSV challenge [33,34]. An important mechanism for regulating immune senescence is autophagy. By removing accumulated proteins and other unnecessary cytoplasmic material, autophagy limits cellular stress and promotes survival of long-lived memory cells [35,36]. Autophagy functions have been shown to decline with age [37,38]. Studies have shown that impairment of key autophagy genes in aged mice or by a genetic deletion system in young adult mice leads to loss of function in memory CD8 T cells and enhanced production of inflammatory cytokines including interferon (IFN)-γ, interleukin (IL)-1β, tumor necrosis factor (TNF), and IL-6 [38,39,40,41,42]. Thus, impaired autophagy mechanisms in elderly individuals likely contribute to the inflammaging phenotype. A recent study demonstrated that supplementation with the metabolite spermidine enhanced the function of T cells isolated from elderly adults by increasing autophagy level and function [38]. This suggests that designing an RSV vaccine that can restore autophagy functions may be an effective strategy for providing protection in an aged population. 

### 2.2. Defects in Cellular Immunity

As an individual ages, the production of new T cells entering the periphery from the thymus declines and the homeostatic proliferation of existing memory T cells decreases [43,44,45]. Thus, the immune system increases in predominantly dysfunctional and terminally differentiated memory cells [46]. Additionally, activation of T cell receptor signaling is impaired in otherwise healthy aged individuals compared to young adults [47]. These defects result in a diminished ability to mount robust T cell responses to new as well as previously encountered pathogens.

Virus-specific memory T cells correlate with protection from RSV infection and are critical for reducing disease pathogenesis, making them an important population to induce through vaccination [48,49,50]. RSV-specific cellular immune responses are impaired in both their number and functional capacity in elderly adults. In vitro stimulated human peripheral blood mononuclear cells (PBMCs) from adults >65 years old were deficient in RSV-specific IFN-γ production compared to adults age 20–40 [51,52]. Similarly, aged mice exhibited a reduced frequency of RSV M2_82–90_-specific cells in the lung and a decreased capacity to produce IFN-γ compared to young mice [53]. The number of RSV-specific memory CD8 T cells have also been shown to diminish with age in humans [54,55]. In contrast, one study found an increase in the total frequency of activated (CD38^+^HLA-DR^+^) CD8 T cells in the blood of older persons with severe RSV-associated disease compared to individuals with mild disease [56]. This suggests that increased activation of CD8 T cells may be implicated in causing enhanced disease. This is supported by murine studies showing that an elevated memory CD8 T cell response in the absence of an antibody response leads to enhanced disease [57]. However, the previous human study failed to assess the specificity or the functional capacity of the T cells, thus the role of RSV-specific CD8 T cells in disease pathogenesis in elderly adults remains unclear [56]. Overall, impaired virus-specific cellular immune responses in elderly adults should be considered when developing a vaccine to target this population.

### 2.3. Dendritic Cell Impairments

Impairments in dendritic cells (DCs) are observed in aged individuals. Aged humans exhibit numerical declines in specific subsets of DCs compared to healthy young adults [58]. DCs isolated from elderly adults are functionally impaired, exhibiting reduced production of IFN-α in response to influenza infection and other antigens [58,59,60,61]. IFN-α is critical for initiating early antiviral responses against RSV [62,63]. Proinflammatory cytokines including IL-6 and TNF are also produced at lower levels by aged primary human DCs in response to various Toll-like receptor (TLR) stimuli [60]. This coincides with a reduced expression of TLRs on the surface of aged DCs isolated from PBMCs [58]. DC interactions with T cells are critical for the priming and subsequent activation of naïve T cells. DCs from adults >65 years old demonstrated a reduced ability to stimulate naïve CD4 and CD8 T cells as measured by reduced proliferation, cytotoxicity, and cytokine production, as well as the reduced presentation of ovalbumin (OVA) antigen on major histocompatibility complex class I (MHC-I) [61,64,65,66]. While studies looking at defects in aged DCs during RSV infection are limited, reports on influenza have demonstrated that DCs from aged mice show delayed migration kinetics into the lungs and lung draining lymph nodes following infection [67,68]. These studies suggest that the functional alterations in aged DCs may affect the priming of T cells, inhibiting the generation of a vaccine-specific immune response.

### 2.4. Defects in Humoral Immunity

The aged humoral immune response is characterized by a decline in the production of new naïve B cells from the bone marrow [69]. The human peripheral B cell pool becomes dominated by class-switched B cells, resulting in diminished replenishment of the circulating B cell clonal repertoire and reduced diversity [70]. Somatic hypermutation and class-switch recombination, which are essential for the generation of isotype-switched antibody responses against encountered pathogens as well as vaccinations, are also impaired in aged mice [71,72]. Overall, this results in a reduced ability to recognize and respond to new antigens. In elderly humans, RSV-specific antibody responses including serum neutralizing antibody titers have been shown to diminish with age [20,21,73]. This low neutralizing titer correlates with an increased risk of developing severe disease following RSV infection [20]. In addition, low serum neutralizing antibody titers may predispose elderly adults to RSV infections, as individuals with higher pre-existing neutralizing titers against either RSV A or B strains were less likely to be infected during two RSV seasons [74,75]. Overall, these data suggest that the cellular immune response in the elderly is characterized by alterations in functional effector capacity and a diminished ability to efficiently respond to antigen stimulation, while the humoral immune response is characterized by a reduced neutralizing capacity.

## 3. Designing a Vaccine to Target Elderly Adults

### 3.1. Optimum Vaccine Subtype

Elderly adults pose the challenge of possessing varying levels of pre-existing immunity to RSV. Any pre-existing immunity may impact the immune response to a vaccine [76]. The use of a live-attenuated vaccine in this population is therefore less ideal, as its effectiveness may be diminished by virus-specific cellular and humoral immune responses [77]. One study in adults >60 years old demonstrated that the inactivated influenza vaccine had a higher efficacy (49–65%) compared to the live-attenuated formulation (3–20%). While it was not directly addressed, the authors speculate that the poor response of the live-attenuated vaccine may be driven by pre-existing immunity to influenza, as a lower efficacy was also observed in healthy adults previously exposed to influenza [78]. Similarly, a recombinant herpes zoster subunit vaccine demonstrated superior protection compared to the currently approved live-attenuated formulation in adults >50 years old [79]. These data suggest that subunit and vector-based modalities will likely be the most efficacious formulation for this population. These vaccines also allow for increased antigen dose compared to whole virus vaccines, a likely requirement to achieve protection in an elderly population. The efficacy of vaccine subtypes in a pre-immune population remains an under studied area of research and should be carefully considered when designing a vaccine for an elderly population. 

### 3.2. RSV Target Antigens

Studies looking at both basic RSV immunology and vaccine-specific responses have provided some insight into the factors that are critical for a robust RSV-specific immune response. Our increased understanding about the structure of the RSV F protein have led to the development of novel, highly immunogenic F protein variants for use in vaccines [80]. The RSV F protein is located on the surface of the virion and functions to mediate viral fusion and entry into host cells, making it a major vaccine target [81,82]. The F protein exists in multiple conformations, a prefusion structure prior to virus-cell fusion and undergoes a conformational change to a postfusion state after fusion occurs [83]. The prefusion structure possess unique antigenic sites not present on the postfusion structure, and importantly, the majority of neutralizing antibody activity found in human serum is attributed to antibodies that target epitopes only exposed on the prefusion conformation [84,85]. The inclusion of the postfusion F protein in early vaccine candidates likely contributed to their failure to generate robust efficacy in an aged population [86,87]. Thus, the prefusion structure is an important immunological target for vaccine design.

In addition to the F protein, including an internal conserved viral protein such as the nucleocapsid (N), matrix (M), or M2–1 protein may help increase the breadth of the RSV-specific T cell response and induce a more robust Th1 response following vaccination. A Modified Vaccinia Ankara vector-based vaccine (MVA-BN-RSV) containing RSV F as well as the N and M2 proteins exhibited promising phase II results in older adults [88]. While the study did not evaluate the additive benefit of including the N or M2 proteins, there was a measurable T cell response specific for both proteins. Additional vaccines including the N and/or M2 proteins have demonstrated protein-specific T cell responses when tested in healthy young adults [89,90,91]. However, no formulation undergoing current evaluation has tested whether a T cell response is required for protection, or examined the efficacy of these vaccine candidates in an older population. Thus, the potential immunogenic benefit of additional RSV antigens in an elderly vaccine warrants further investigation.

### 3.3. Overcoming Immune Senescence

Understanding how to overcome immune senescence will be critical for developing a successful vaccine for preventing RSV infection in elderly adults. One strategy to enhance the immunogenicity of a vaccine is the addition of one or more adjuvants. Adjuvants can enhance or skew the immune response to a vaccine antigen by acting on the primary innate sensing response and subsequently influencing the developing adaptive immune response. Oil-in-water emulsions such as MF59 and AS03 are currently approved for use in influenza vaccines for older adults. These adjuvants act independently of TLR signaling, activating antigen presenting cells at the site of injection to produce chemokines such as CCL2, CCL3, and IL-8, enhancing immune cell recruitment and further subsequent antigen uptake and transport [92,93,94]. In clinical trials, MF59-adjuvanted vaccines administered to the elderly demonstrated increased efficacy at preventing hospitalizations due to influenza, as well as influenza-associated pneumonia compared to the standard influenza vaccine [95]. A polymer nanoparticle-based vaccine, termed ResVax, composed of RSV F trimers demonstrated safety and efficacy in phase I and II clinical trials [86]. However, it failed to meet its primary endpoint in a 2015 phase III clinical trial (RESOLVE trial) in older adults [96]. In a follow-up phase II trial, the inclusion of an aluminum phosphate (alum) adjuvant increased the magnitude and quality of the immune response compared to the non-adjuvanted vaccine [97]. Although alum has been used extensively in humans and no significant adverse events related to alum were reported in the phase II trial, studies in animal models suggest caution may be warranted when considering the use of alum combined with RSV antigens. The use of alum as an adjuvant combined with the RSV F protein has been shown to increase lung pathology in vaccinated mice challenged with RSV despite mediating viral clearance [98]. This effect occurs despite formulation with the prefusion F protein and the generation of high-quality neutralizing antibodies. This highlights the importance of including the appropriate adjuvant to help the vaccine overcome the limitations of the aged immune system.

Additionally, there are numerous TLR-dependent adjuvants approved for use in humans, and more specifically in elderly adult populations. The liposomal adjuvant AS01, which is a combination of the TLR4 agonist 3-O-desacyl-4′-monophosphoryl lipid A and QS-21 is currently licensed and used in an elderly vaccine for varicella-zoster virus (herpes zoster) [99]. An AS01-adjuvanted subunit herpes zoster vaccine induced 10-fold higher CD4 and CD8 memory T cells compared to a live-attenuated virus strain in adults 50–85 years old [100]. Another TLR4 agonist glucopyranosyl lipid A (GLA), demonstrated immunogenicity in preclinical studies, enhancing granzyme B and IL-6 production in PBMCs from adults >60 years old in response to influenza antigen [101,102]. GLA has more recently been tested in an RSV vaccine containing the postfusion conformation of the RSV F protein. This vaccine demonstrated safety and immunogenicity in a phase I clinical trial in adults >60 years of age [103]. However, it failed to prevent RSV-associated acute respiratory illness in a phase II clinical trial in the same population [87]. The vaccine was administered as a single dose which may not provide enough of a boost to pre-existing immunity to provide protection in this population [104,105]. Additionally, the failure of this vaccine candidate may have been due to the antigen selection rather than the ineffectiveness of the adjuvant. Follow-up studies in aged mice utilizing the prefusion structure of RSV F combined with GLA demonstrated the induction of enhanced neutralizing antibody titers and increased protection compared to both the postfusion-based formulation and unadjuvanted controls [106]. Thus, determining the most efficacious antigen and adjuvant combination in preclinical studies will be critical for informing future clinical trials.

Another strategy to help compensate for the immunological impairments of the aged immune system is to increase the antigen load. In both phase II and III clinical trials, a four-fold increase in influenza hemagglutinin antigen significantly increased the serum virus-specific antibody responses in vaccinated adults >65 years of age, without enhancing vaccine-related side effects [107,108,109,110]. Another phase I study assessing an RSV F particle-based vaccine in elderly adults demonstrated that a 90 μg dose of antigen significantly increased the titer of RSV F-directed antibodies and neutralizing antibody titers compared to a 60 μg dose [86]. Although the inclusion of an aluminum phosphate adjuvant also increased the titer of antibody, suggesting again that both aspects are important for vaccine design. Several other phase I clinical trials in adults >60 years old have demonstrated significant increases in RSV-specific antibody titers with increasing antigen dose [103,111]. 

### 3.4. Vaccine Candidates in Clinical Trials

To date, there are no RSV vaccines that have successfully demonstrated robust efficacy in older adults. However, there are several candidates currently undergoing evaluation in clinical trials (Table 1) [112,113]. MVA-BN-RSV, a non-replicating vector-based vaccine based on the Modified Vaccinia Ankara (MVA) backbone, demonstrated safety in a phase I clinical trial in adults (50–65 years old) [88]. Successful phase II results revealing robust neutralizing antibody titers and a broad Th1-biased T cell response have led to subsequent plans for an upcoming phase III trial [105]. A messenger ribonucleic acid (mRNA)-based vaccine encoding the prefusion F protein was recently tested in a phase I clinical trial in adults 60–80 years old [114]. The vaccine demonstrated safety and tolerability, and elicited RSV-specific serum neutralizing antibodies. Of note, two mRNA vaccines for SARS-CoV-2, BNT162b2 from Pfizer-BioNTech and mRNA-1273 by Moderna, recently demonstrated efficacy in elderly adults in phase III clinical trials [115,116,117]. Compared to healthy young adults, adults >60 years of age demonstrated similar antibody responses across multiple doses, suggesting the potential of the mRNA vaccine platform to elicit protective immunity in this population [118]. These recently became the first clinically approved mRNA-based vaccines.

Several groups have developed adenovirus vector-based vaccine candidates. Recombinant adenovirus vectors allow for the generation of robust Th1-skewed immunity and can possess intrinsic adjuvant properties which are critical for targeting an adult pre-immune population. However, individuals can have pre-existing immunity against some serotypes of adenoviruses, which may dampen the vaccine-induced response [119,120]. A human adenovirus expressing a stabilized prefusion F (Ad26.RSV.Pre-F) demonstrated safety and immunogenicity when administered as a prime-boost to healthy adults >60 years in a phase I clinical trial [111,121,122]. Recently, Ad26.RSV.preF was found to significantly reduce RSV viral load and disease severity compared to placebo in an experimental RSV challenge model in healthy young adults [123]. While this study demonstrates the potential of this vaccine candidate, phase III clinical trials are needed to verify these findings and more firmly establish the efficacy of this vaccine platform in an elderly target population. 

Subunit vaccines containing RSV proteins are non-replicating and require the inclusion of one or more adjuvants to drive the most beneficial immune response. GSK3844766A, a platform containing a recombinant prefusion RSV F antigen with an AS01 adjuvant elicited robust RSV F-specific CD4 T cells and protective antibody responses in elderly adults [124]. However, results of this trial have not yet been published. Overall, there are a variety of novel vaccine candidates currently in testing, and the results from these trials will serve to inform the design of future vaccines for use in the elderly.

## 4. Future Perspectives

Immune senescence has been shown to negatively impact many of the factors that are required for an effective RSV vaccine, including the induction of memory T cells and class-switched antibody responses. A vaccine designed to target elderly adults will need to maximize the host immune response in order to overcome these defects. The best strategies for boosting vaccine-induced responses in this population are to select the most potent RSV antigen(s), identify the optimal antigen load, and utilize an appropriate adjuvant. The inclusion of multiple viral proteins may increase the RSV-specific T cell response and sustain the lifespan of these important populations. Additionally, the antigen quantity provided in a single-dose vaccine may not be sufficient to generate enough of a boost in an already less responsive immune system to provide long-term protection. A higher antigen dose delivered over one or more booster vaccinations may enhance RSV-specific antibody responses in older adults. An important consideration particularly for an elderly vaccine will be determining the best method and formulation to deliver an optimal antigen dose. In addition, while the mortality rates are high in elderly individuals, the overall rates of RSV infection in a given year remain relatively low. Indeed, several unsuccessful clinical trials speculate that they failed to meet their primary endpoints due to an overall low frequency of natural RSV infection throughout the study [87,125]. Thus, larger scale clinical trials with higher power need to be designed to confidently determine the accurate vaccine efficacy even during low infection years. RSV exhibits seasonality in most locations, with cases peaking during the winter season. The duration of protection afforded by a vaccine should be sufficient to get an individual through an entire season.

There is still much about the aging immune system that remains unknown. While studies in aged mice have shed light on some aspects of the deficits associated with aging, it will likely be difficult to ascertain all of the shortcomings in an animal model. In particular, homeostasis is regulated differently between mice and humans, and the timescale of aging may result in different impairments [126]. For example, while differences in MHC-I and MHC-II and co-stimulatory molecule (CD80, CD86) expression on DCs are observed in young compared to aged mice, the same differences have not been observed in human studies [65,127,128]. Additionally, assessing immune responses to a natural RSV infection in humans is made difficult by the variability in the studies. Longitudinal studies of community-dwelling adults primarily rely on blood and serum to assess in vitro systemic immune responses prior to and post RSV infection. However, the time elapsed between samples is frequently variable which subsequently weakens the ability to address mechanistic questions regarding the changes observed over time. In addition, systemic responses may not reflect the alterations occurring within the tissues. In the gastrointestinal mucosal tissue, distinct differences are observed in the aged T cell phenotype compared to blood samples from the same individuals [129]. To our knowledge, a comparative analysis of the effects of aging on peripheral blood mononuclear cells and lung tissue has not been performed. While there are still many aspects of the immune response to RSV in older adults that require further study, it is clear that age-related changes in the immune system will likely impact the development of any vaccine-mediated immunity to RSV and should be carefully considered.

## 5. Conclusions

RSV is an important viral pathogen among aging adults, leading to enhanced mortality and increased development of severe RSV-associated symptoms. Much of severe disease as well as the increase in overall hospitalization stay could likely be reduced through the use of vaccines. As aging occurs, the immune system exhibits a progressive decline in function, characterized by dysfunctional memory cells. A vaccine designed to target an elderly population will need to maximize the immune response generated in order to overcome this immune senescence. Studies in aged populations have shown that a number of factors including antigen dose and adjuvant selection play a critical role in amplifying the immune response generated by a vaccine. Additionally, it will be important to choose the best combination of antigens to overcome the deficiencies in the aged immune system. A number of vaccine candidates targeting elderly adults are currently in clinical trials. However, none to date have demonstrated efficacy in protecting against RSV infection. Further research into the mechanism governing the adverse changes that occur in the immune system during aging will be critical for developing a vaccine to exploit or overcome these defects.

## Figures and Tables

**Table 1:** RSV vaccine candidates targeting elderly adults in clinical development.

Vaccine Candidate, Sponsor	Stage of Development	Formulation/Antigen	Results	Reference
mRNA-1777	Phase I	mRNA	Neutralizing antibodies	[114]
Moderna, Merck & Co.		Prefusion RSV F	RSV F-specific serum antibodies	
			RSV-specific CD4 and CD8 T cells	
				
VXA-RSVf	Phase I	Vector-based	N/A	[113]
Vaxart		RSV F		
PanAd3-RSV	Phase I	Vector-based	RSV-specific IgG and IgA	[112]
ReiThera		RSV F, N, M2	T cell mediated IFN-ɣ	
Ad26.RSV.Pre-F	Phase II	Vector-based	Ongoing, N/A	[111,121,122,123]
Janssen		Prefusion RSV F		
MVA-BN-RSV	Phase II	Vector-based	Well tolerated	[88,105]
Bavarian Nordic		RSV F, G, N, M2	Neutralizing antibodies	
			T cell mediated IFN-ɣ	
MEDI-7510	Phase II	Subunit	RSV F-specific serum IgG	[87,103]
MedImmune		Postfusion RSV F + GLA-SE	RSV F-specific IFN-ɣ	
			Did not meet primary endpoint	
GSK3844766A	Phase III	Subunit	Ongoing, N/A	[124]
GlaxoSmithKline		Prefusion RSV F + AS01		
ResVax	Phase III	Particle-based	Acceptable safety and tolerability	[86,96,97]
Novavax		RSV F trimers	RSV-specific serum IgG	
			Neutraliizing antibodies	
			Did not meet primary endpoint	

## Data Availability

Not applicable.
